# Performance of CAD4TB artificial intelligence technology in TB screening programmes among the adult population in South Africa and Lesotho

**DOI:** 10.1016/j.jctube.2025.100540

**Published:** 2025-06-04

**Authors:** Nonhlanhla Nzimande, Keelin Murphy, Klaus Reither, Shannon Bosman, Irene Ayakaka, Tracy R. Glass, Fiona Vanobberghen, Bart K.M. Jacobs, Aita Signorell, Jabulani Ncayiyana

**Affiliations:** a Division of Public Health Medicine, School of Nursing and Public Health, College of Health Sciences, University of KwaZulu-Natal, Durban, South Africa; b Centre for Community Based Research, Human Sciences Research Council, Pietermaritzburg, South Africa; c Radboud University Medical Center, Nijmegen, the Netherlands; d Swiss Tropical and Public Health Institute, Allschwil, Switzerland; e University of Basel, Basel, Switzerland; f Liverpool School of Tropical Medicine, Liverpool, United Kingdom; g Department of Clinical Sciences, Institute of Tropical Medicine, Antwerp, Belgium

**Keywords:** CAD4TB, Artificial intelligence, TB screening, chest X-rays, Algorithm

## Abstract

There is growing evidence of the performance accuracy and potential impact of Computer-Aided Diagnosis (CAD) products in TB-burdened settings. It remains unclear, however, which factors of populations and settings can affect CAD performance. We aimed to investigate the parameters affecting the performance accuracy of the two latest versions of CAD4TB in TB screening programmes in South Africa and Lesotho.

We included participants recruited for the Lesotho National Prevalence Survey and the TB TRIAGE + ACCURACY studies, who underwent digital chest radiography and microbiological reference testing for TB. In total, 6,524 chest images were included in the analysis: 288 cases and 6,236 controls. CAD4TB versions 6 and 7 interpreted the X-ray images, and the performance of both versions was investigated. Threshold analyses were performed, as well as subgroup analyses, including age, X-ray hardware and HIV status.

CAD4TB v7 showed overall improved performance accuracy compared to v6 (p < 0.01). The area under the ROC curve was 0.833 (95 % CI 0.808–0.859) for v6 and 0.865 (95 % CI 0.842–0.889) for v7. At 90 % sensitivity, v7 showed a higher specificity of 65 % compared to the 54 % achieved by v6. Both versions showed lower performance in the older age group (≥60 years) and individuals with a previous history of TB. The threshold required to achieve the same sensitivity or specificity varies notably across the two versions.

CAD4TB performed well as a TB screening tool; however, factors such as software version, age, TB history and X-ray hardware should be considered in threshold determination and performance evaluation.

## Highlights

• CAD4TB version 7 significantly outperformed version 6.
• Both versions showed decreased performance in older individuals and people with a previous history of TB.
• Variability noted across the different X-ray hardware systems used.
• Thresholds differed across the two versions, and the various demographic and characteristic subgroups.
• CAD4TB version 7 is closer to meeting the WHO Target Product Profile’s recommendation for triage test.

## 1 Introduction

Tuberculosis (TB) remains a significant public health concern despite being preventable and curable. In 2023, 10.8 million (95 % CI 10.1 – 11.7 million) people developed TB globally, with over 2.7 million out of the reported 10.8 million being undiagnosed.[1] There is an urgency to achieve early diagnoses, which lead to better patient outcomes[2,3] and to eradicate TB by 2030.[4] Tests such as mycobacterial culture.

or Xpert MTB/RIF Ultra (GeneXpert, Cepheid, Sunnyvale, CA, USA) are the most accurate confirmatory tests for active TB.[5,6] Still, delayed turnaround times, high operational costs and limited accessibility in resource-constrained TB-burdened populations remain a significant concern.[7,8] Usage of chest X-rays to screen for TB has shown high sensitivity when compared to symptom screening and is cost-effective.[9] TB-related lung abnormalities can resemble other lung pathologies, which limits the specificity of chest X-rays.[10] A standard model is to screen using chest X-ray and only use bacteriological tests on those with suspicious abnormalities.[11] A limiting factor in using chest X-rays as an effective TB screening tool is the need for more radiologists in resource-constrained populations.[12] In 2021, the WHO endorsed using artificial intelligence (AI) products for chest X-rays in TB screening.[13] The WHO Target Product Profile recommends that a triage test have a minimum sensitivity and specificity of 90 % and 70 %, respectively.[14] With over 15 commercially available CAD programmes for TB screening,[15] many never evaluated in the literature[16], there is renewed research interest in using chest X-rays with artificial intelligence (AI) as a fast, effective, minimally invasive and easily accessible TB diagnostic tool.

AI algorithms are trained using large datasets of labelled data to recognise patterns and interpret images without human interference.[[17], [18], [19]] The CAD4TB (Delft Imaging, ‘s-Hergotenbosch, the Netherlands) product is one of several available Computer-Aided Detection (CAD) programmes that use AI technologies to recognise pulmonary TB-related findings on chest radiographs.[8,14] The CAD4TB version 6 was released in 2018,[8] followed by version 7 in 2021 as an improvement to v6.[20] Studies have demonstrated that version 7 outperforms version 6 overall (higher AUC), with improved specificity[21,22] and greater cost effectiveness as a TB screening tool.[21,23,24].

The programme is trained to detect TB-related abnormalities automatically in the image, assigning a continuum abnormality score (CAD score) ranging from 0 to 100.[20,21]. Operators should proceed with confirmatory bacteriological testing if the score exceeds a certain preset threshold set according to their requirements [3,25] Research has also shown CAD4TB capabilities to perform on par or outperform human radiologists, a significant advantage in resource-constrained and TB-burdened settings like South Africa and Lesotho.[8,21] Several studies have also demonstrated the inter-version variation with different distributions of CAD score, emphasising threshold optimisation.[21,22,24,26] However, more literature on African populations and threshold-specific CAD analysis, including threshold differences between hardware systems, is still needed to expand its application. We investigated five different hardware systems used in the two studies and explored the optimal thresholds for each hardware.

Without any clear guidelines for users on selecting thresholds specific to their settings, there is a danger of missing actual TB-positive individuals or wasting resources by performing confirmatory testing on a high proportion of true TB-negative individuals.[9,11] We aimed to investigate and compare the diagnostic accuracy of the two latest CAD4TB software versions (v6 and v7) on chest X-rays in screening for TB among the adult population in South Africa and Lesotho, using Xpert MTB/RIF ultra and culture as reference standards. Furthermore, the analysis of CAD4TB performance was stratified in multiple ways, including software version, participant age, gender, HIV status, country, previous TB, and hardware used in image acquisition. All data used in this study is known to be fully independent of the training data used for the CAD4TB products.

## 2 Methods

### 2.1 Study design

This retrospective case-control diagnostic accuracy study used digital chest X-rays and data from individuals who participated in one of the studies (TB TRIAGE + ACCURACY study [Clinicaltrials.gov identifier: NCT04666311] and Lesotho TB National Prevalence Survey) between February 2021- April 2022 and March- November 2019, respectively.[27,28] The CAD4TB software and images were provided to Radboud University Medical Center (UMC) and run offline by a researcher involved in this work (KM). No preprocessing was applied to the images; they were supplied to the CAD4TB software, and the output scores were recorded.

### 2.2 Study population and study setting

The TB TRIAGE + ACCURACY (TBT + ) study[28] was a prospective two-centre cross-sectional study recruiting patients in Lesotho and South Africa healthcare settings. The study enrolled 1392 individuals aged 18 years and older who presented with TB symptoms for any duration. All participants had a digital chest X-ray and were asked to provide two sputum specimens irrespective of chest X-ray findings. The samples were tested using Xpert MTB/RIF Ultra (Xpert; Cepheid, USA) and culture (BACTEC MGIT 960; Becton Dickinson, USA). The precise definitions of how TB-positive and TB-negative cases were defined can be found in the study publication[28]. Following the exclusions detailed in Fig. S1 (supplementary material), 172 individuals were excluded, and 1,220 were included in our study population.

The Lesotho TB National Prevalence Survey (LPS)[27] was a multi-stage cluster-based cross-sectional survey that included all individuals aged 15 years and above. In total, 39,902 individuals were enumerated across 15,279 households. After being screened using a chest X-ray, individuals with either chest X-ray abnormalities, who reported TB symptoms, or who refused a chest X-ray were asked to produce two sputum samples tested using Xpert MTB/RIF Ultra and culture, respectively. The precise details of how TB-positive and TB-negative cases were defined are described in the study publication.[27].

Despite best efforts, not all images from the LPS could be obtained for our evaluation. Thus, 1,236 participants were excluded as a chest X-ray was unavailable. A further 33,284 had no microbiological test result since this was conditional on pre-screening. Following other exclusions detailed in Fig. S1, S5,304 (13.29 %), participants were included in our study population.

### 2.3 Statistical analysis

All cases and controls from the two datasets were included in the study to increase the statistical power in subgroup analysis. To compare the performance of the newer version of CAD4TB (v7) against its predecessor, we conducted a threshold-independent analysis by plotting the non-parametric receiver operating characteristic (ROC) curve for each version using microbiological reference standards. The area under the ROC curve (AUC) was then calculated with 95 % confidence intervals. DeLong’s algorithm [29] with p < 0.05 was used to determine statistical significance.[30].

A threshold-dependent analysis was conducted to investigate the impact of different thresholds on the performance of the two versions [31]. The sensitivities and specificities of each version were plotted per threshold value. The performance of CAD4TB at a sensitivity of 90 % and at a specificity of 70 % is reported to provide sample operating point information.

We performed subgroup analyses to investigate whether the CAD4TB performance varies across different populations. The study population was stratified by age groups (15–35, 36–60, 61 + ), sex (male, female), HIV status (HIV negative, HIV positive), previous TB history (no previous TB, had TB previously), country (South Africa, Lesotho), study name (TBT+, LPS) and X-ray hardware used in image acquisition (Delft Light Portable X-ray machine, FUJIFILM FDR Smart X-ray machine, Innomed X-ray machine, Sedecal Dragon 5 kW Digital X-ray machine). The performance of each version of CAD4TB was analysed in the same way as the overall analysis described above. The optimal threshold required for each group to achieve a 90 % sensitivity and 70 % specificity was estimated and compared. Statistical testing of the equality of the two ROC areas from two independent samples was done for each subgroup using Stata “*roccomp (long-form)”*.[30] We considered a p-value of less than 0·05 statistically significant. Bonferroni multiple testing correction was conducted in cases with more than two subgroup categories.

All statistical analyses were done with Stata version 18 (StataCorp. 2023. *Stata Statistical Software: Release 18*. College Station, TX: StataCorp LLC) (StataCorp.

### 2.4 Ethical considerations

All images were anonymised and transferred securely to Radboud UMC and not stored in the cloud for the purpose of this study.

Ethical approval was obtained from the University of KwaZulu-Natal Biomedical Research and Ethics Committee, reference number BREC/00006739/2024. For the TB TRIAGE + ACCURACY study, the following ethical approvals were obtained: The Northwest and Central Switzerland Ethics Committee (AO_2022-00014), the National Health Research and Ethics Committee of Lesotho (ID 100–2020), the Human Sciences Research Council Research Ethics Committee (REC 2/23/09/20), and the KwaZulu Natal Provincial Department of Health Research Ethics Committee (KZ_202102_030) in South Africa.

Ethical approval for the Lesotho National Prevalence Survey was obtained from the Lesotho Research and Ethics Committee (ID 23–2017, June 29, 2018).

The anonymised datasets from both parent studies were supplied through the TB TRIAGE + consortium and stored in a password-protected device secured with a scheduled backup and restoration.

### 2.5 Role of the funding source

The study's funders had no role in the design, data collection, analysis, interpretation, or report writing.

## 3 Results

Table 1 includes details of participants' demographics and characteristics stratified by TB status. Of the 6,524 participants included in our study population, 288 (4 %) were TB-positive, bacteriologically confirmed (cases), and 6,236 (96 %) were TB-negative, bacteriologically confirmed (controls). The flow chart showing the selection of participants for the study is shown in Fig. S1 (supplementary material). Tables S1 and S2 (supplementary material) show the participants' demographics per study.

**Table 1:** Baseline characteristics.

	**TB negative 6 236, (95.59 %) N, (column %)**	**TB positive 288, (4.41 %) N, (column %)**	**Total (n 6 524) N, (column %)**
**Age (yrs.) Mean, SD; median IQR**	49, 18.63**;** 50, 30	48, 17.61**;** 46, 28.87	49, 18.59**;** 50, 30
**Age group**			
15 to < 35 years 1 592 (25.53) 78 (27.08) 1 670 (25.60)			
35 to < 60 years	2 571 (41.23)	122 (42.36)	2 693 (41.28)
≥60 years	2 073 (33.24)	88 (30.56)	2 161 (33.12)
**Sex**			
Male	3 139 (50.34)	191 (66.32)	3 330 (51.04)
Female	3 097 (49.66)	97 (33.68)	3 194 (48.96)
**Country**			
South Africa	477 (7.65)	65 (22.57)	1 220 (18.70)
Lesotho	5 759 (92.35)	223 (77.43)	5 304 (81.30)
**Study enrolled to**			1 220 (18.70)
a TB TRIAGE+	1 086 (17.42)	134 (46.53)	5 304 (81.30)
b LPS	5 150 (82.58)	154 (53.47)	
**HIV status**	4 075 (65.35)	143 (49.65)	4 218 (64.65)
HIV negative	1 673 (26.83)	122 (42.36)	1 795 (27.51
HIV positive	488 (7.83)	23 (7.99)	511 (7.83)
Unknown	609 (9.77)	69 (23.96)	678 (10.39)
c**Hardware**	477 (7.65)	65 (22.57)	542 (8.31)
Delft	1 842 (29.54)	48 (16.67)	1 890 (28.97)
Fujifilm	2 436 (39.06)	89 (30.90)	2 525 (38.70)
Innomed1	872 (13.98) *−*	17 (5.90)	889 (13.63)
Innomed2 Sedecal	5 146 (82.52)	230 (79.86)	5 376 (82.40)
**Previous TB**	1 090 (17.48)	58 (20.14)	1 148 (17.60)
No	52.19 (45,59)		53.26 (45, 61)
Yes	20.04 (3, 31)	76.47 (63, 92.5)	21.88 (3, 34)
**CAD4TB**		27.36 (42.46, 85.85)	
d v6 −median (Q1, Q3)			
e v7 −median (Q1, Q3)			

= Delft Light Portable X-ray machine with Canon detector, CXDI Control Software; Fujifilm.

=FUJIFILM FDR Smart X-ray machine with Vieworks detector, FXRD-1717VA; Innomed1 = Innomed X-ray machine with Samsung detector, SDR-AGR40CW: SMD4343WS; Innomed2 = Innomed X-ray machine with Samsung detector, DGR-RN2N22/WR: SMD4343WS; Sedecal =Sedecal Dragon 5 kW Digital X-ray machine with integrated detector.

a TB TRIAGE + ACCURACY study.

b LPS (Lesotho Prevalence Survey).

c Hardware (hardware used in image acquisition) Delft.

d CAD4TB v6 (CAD abnormality score).

e CAD4TB v7 (CAD abnormality score).

Most of the participants in our study population, 5,376 (82.40 %), did not have a previous history of TB. CAD4TB v6 showed a higher abnormality score, with a median of 53.26; Q1, Q3 (45, 61) compared with CAD4TB v7, with a median of 21.88; Q1, Q3 (3, 34).

### 3.1 Overall performance analysis

**Fig. 1:**
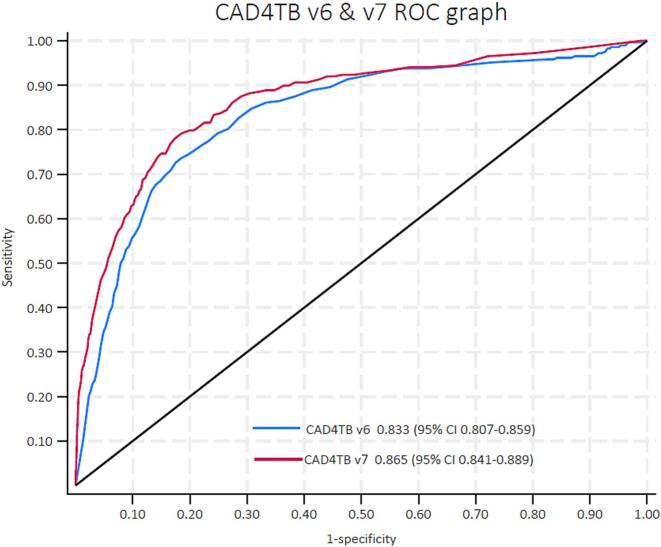
**Threshold independent analysis**: Comparing CAD4TB v6 and CAD4TB v7 receiver operating characteristic (ROC) using a bacteriological reference standard The area under the ROC curve is shown per CAD4TB version, followed by the 95 % confidence interval for this value.

Fig. 1 shows the ROC curve for each version's overall performance against bacteriological reference standards with confidence intervals (CI). The AUC for CAD4TB v7 was 0.87 (95 % CI 0.84–0.89), slightly surpassing that of CAD4TB v6, which is 0.83 (95 % CI 0.81–0.86). This difference is statistically significant (p < 0.01). Fig. S2 (supplementary material) shows a threshold-independent analysis conducted for the two datasets separately (TBT + and LPS) to account for any variability; again, in each dataset, CAD4TB v7 showed a statistically significantly better performance compared to version 6 (p < 0.01).

**Fig. 2:**
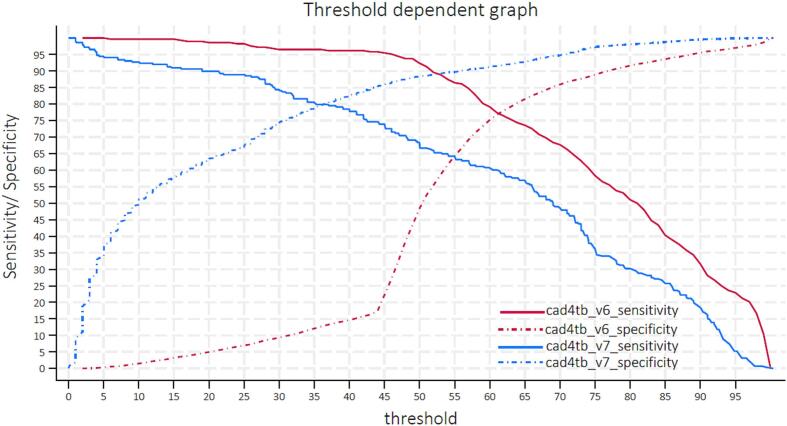
**Threshold dependent analysis:** An illustration of how performance differed per threshold for the two versions of CAD4TB.

A threshold-dependent analysis (Fig. 2) showed that CAD4TB v6 and CAD4TB v7 show very different score distributions. This demonstrates that very different thresholds will be required to achieve comparable performance levels in each version. A threshold-dependent analysis was conducted separately for the TBT + and LPS, showing similar results (Fig. S3).

At 90 % sensitivity, versions 6 and 7 achieved a specificity of 55 % and 65 % at thresholds of 52 and 21, respectively. Subsequently, at 70 % specificity, the sensitivity for version 6 was 83 % at a threshold of 58 and 88 % for version 7 at a threshold of 26 (Fig. 2).

**Fig. 3:**
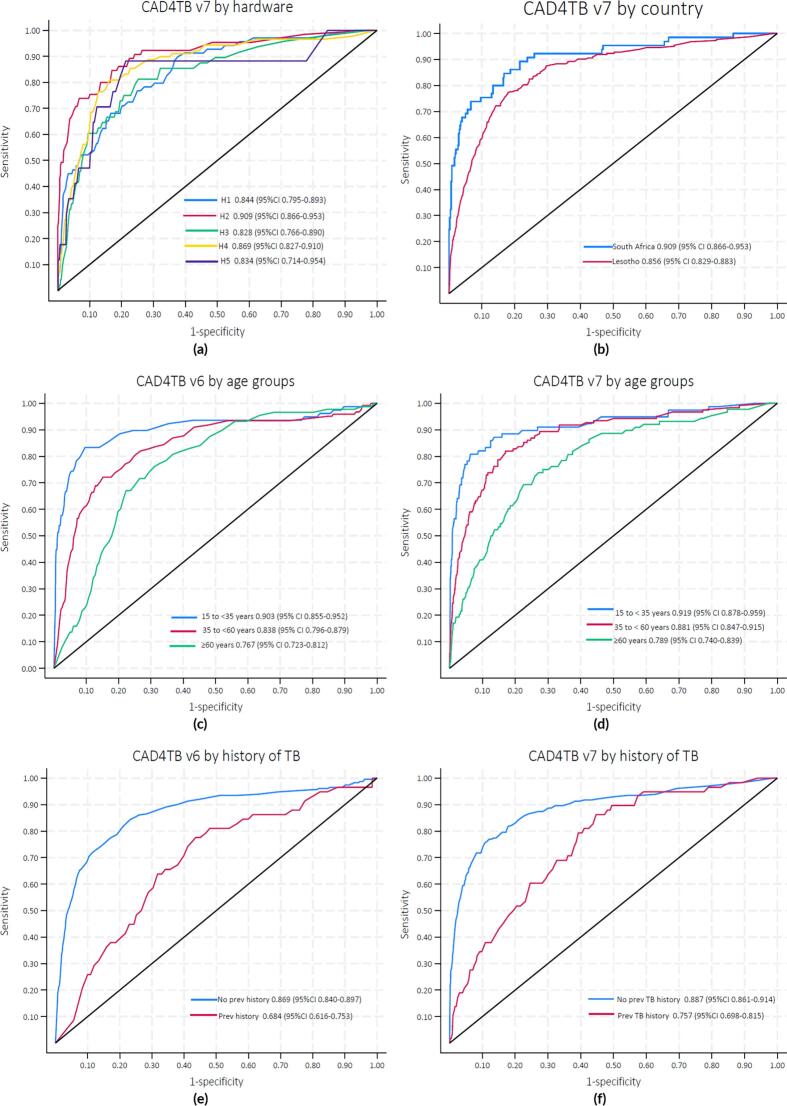
**Sub-group analysis**: Receiver Operating characteristic (ROC) graphs showing subgroups where any statistically significant differences in performance were found. The area under the ROC curve values are shown per CAD4TB version, and confidence intervals are shown.

Table 2 shows complete results for the subgroup analysis, including the area under the ROC curve (AUC) per subgroup, the thresholds needed for 90 % sensitivity or 70 % specificity and the occasions on which a statistically significant difference was found between subgroups. Where significant differences were detected between subgroups for a specified CAD4TB version, the ROC plots are shown in Fig. 3. The ROC plots where significant differences were not found between the subgroups are shown in the supplementary material (Fig. S4). A pairwise comparison of groups with more than two subgroups is shown in Table S3 (supplementary material).

**Table 2:** Subgroup threshold and performance analysis.

				**CAD4TBv6**					**CAD4TBv7**			
		**n**		**AUC (95 %CI)**	**Outperforms (p < 0.05)**	**T1**	**T2**		**AUC**	**Outperforms (p < 0.05)**	**T1**	**T2**
**Overall**		6524		0.83 (0.81–0.86)	−	52	57		0.87 (0.84–0.89)	v6 (p < 0·01)	22	27

**Subgroup Type**	**Group Name**	**n**		**AUC**	**Outperforms (p < 0.05)**	**T1**	**T2**		**AUC**	**Outperforms (p < 0.05)**	**T1**	**T2**
**Hardware**	**H1**	678		0.81 (0.76–0.86)	−	53	59		0.84 (0.79–0.89)		28	35
	**H2**	542		0.85 (0.80–0.91)	−	51	58		0.91 (0.87–0.95)	H3 (p = 0.04)	29	21
	**H3**	1890		0.83 (0.78–0.87)	−	54	56		0.83 (0.77–0.89)	−	7	21
	**H4**	2525		0.84 (0.79-0.089)	−	52	57		0.87 (0.83–0.91)	−	26	27
	**H5**	889		0.77 (0.64–0.89)	−	53	59		0.83 (0.71–0.95)	−	39	33
**HIV Status**	**Neg**	4218		0.84 (0.80–0.88)	−	50	56		0.86 (0.82–0.90)	−	9	25
	**Pos**	1795		0.81 (0.77–0.85)	−	52	59		0.87 (0.84–0.90)	−	29	29
**Age groups (years)**	**15 to < 35**	1670		0.90 (0.85–0.95)	35 to < 60 years (p = 0.04) ≥60 years (p < 0·01)	49	48		0.92 (0.88–0.96)	≥60 years (p < 0·01)	19	12
	**35 to < 60**	2693		0.84 (0.80–0.88)	≥60 years (p = 0.02)	51	56		0.88 (0.85–0.92)	≥60 years (p < 0·01)	22	26
	**≥60**	2161		0.77 (0.72–0.81)	−	57	66		0.79 (0.74–0.84)	−	19	38
**History of previous TB**	**No**	5376		0.87 (0.84–0.90)	Yes (p < 0·01)	52	54		0.89 (0.86–0.91)	Yes (p < 0·01)	16	21
	**Yes**	1148		0.68 (0.62–0.75)	−	51	80		0.76 (0.70–0.82)		38	61
**Country**	**South Africa**	542		0.85 (0.80–0.91)	−	51	58		0.91 (0.87–0.95)	Lesotho (p = 0.04)	29	21
	**Lesotho**	5982		0.82 (0.80–0.85)	−	52	57		0.86 (0.83–0.88)	−	19	27
**Study name**	**TB TRIAGE+** **ACCURACY**	1220		0.83 (0.79–0.87)	−	52	59		0.87 (0.84–0.91)	−	27	29
	**LPS**	5304		0.83 (0.79–0.86)	−	52	57		0.85 (0.81–0.88)	−	16	27
**Sex**	**Male** **Female**	3330 3194		0.84 (0.81–0.87) 0.80 (0.75–0.85)	− −	57 49	62 55		0.86 (0.83–0.89) 0.86 (0.81–0.90)	−	29 7	34 18

**T1** is the threshold that achieves 90% sensitivity, and **T2** is the threshold that achieves 70% specificity.

^a^TB TRIAGE + ACCURACY study.

^b^LPS (Lesotho Prevalence Survey).

^c^Hardware (hardware used in image acquisition) Delft = Delft Light Portable X-ray machine with Canon detector, CXDI Control Software; Fujifilm = FUJIFILM FDR Smart X-ray machine with Vieworks detector, FXRD-1717VA; Innomed1 = Innomed X-ray machine with Samsung detector, SDR-AGR40CW: SMD4343WS; Innomed2 = Innomed X-ray machine with Samsung detector, DGR-RN2N22/WR: SMD4343WS; Sedecal =Sedecal Dragon 5 kW Digital X-ray machine with integrated detector.

^d^CAD4TB v6 (CAD abnormality score).

^e^CAD4TB v7 (CAD abnormality score).

*511 participants with an unknown HIV status were excluded.

### 3.2 Subgroup performance analysis

**Hardware:**Fig. 3(a) shows the ROC performance per hardware system for CAD4TB version 7, where a significant difference was found between hardware Fujifilm and Innomed1. However, these differences were no longer significant after adjusting for multiple testing using Bonferroni correction, as shown in the supplementary material (table S4). Other significant differences were not found. For CAD4TB version 7, variability in the thresholds T1 and T2 is observed for different hardware.

**Country:** When stratified by the country where the participants were enrolled, as shown in Fig. 3(b), CAD4TB version 7 showed statistically significant differences in performance between participants residing in South Africa with an AUC of 0.91 compared to those who lived in Lesotho 0.86 (p < 0.05).

**Age:**Fig. 3(c) and 3(d) show the performance by age group for CAD4TB versions 6 and 7, respectively. Both versions showed lower performance for those of higher age. Both versions showed significant performance differences in the 15–35 age group compared to the older age group (p < 0.01). After applying the Bonferroni correction, some comparison age groups were not significant in version 6, as shown in the supplementary material (Table S4).

**Previous TB:** The product's performance on subgroups separated by the history of TB is shown in Fig. 3(d) and 3(e) for versions 6 and 7. Both versions showed significantly worse performance in people with a prior history of TB. For version 6, the AUC is 0.68 for those with previous TB and 0.87 for the rest (p < 0.01). For version 7, the corresponding figures are 0.76 and 0.89 (p < 0.01).

## 4 Discussion

In our study, CAD4TB v6 and v7 demonstrated good performance with an AUC above 0.80, as shown in previous studies.[8,32] Our results showed that the threshold needed to achieve a particular level of sensitivity/specificity is very different for version 6 and version 7, corroborating findings by Fehr et al.[22,26] Neither version met the minimum requirement of 90 % sensitivity and 70 % specificity set by the WHO Target Product Profile,[14] contrary to one study that has shown that the latest version has met these WHO TPP targets, [21] attesting to the variability of CAD performance in different population settings. However, this is likely to be due to the fact that our dataset is not typical screening data, as described in detail in the study limitations.

The WHO CAD toolkit acknowledges that there could be variability within CAD product versions and advocates for operational research before implementing CAD programmes to determine context-specific optimal thresholds.[11] One of the concerns with AI updates is model drift, a process where data used to train AI models may not accurately represent real-world settings, which can decrease performance accuracy if not monitored through continuous feedback and regular retraining with new data.[33,34] Model drift, as well as differences in calibration and post-processing techniques, can cause an updated version of a product to behave differently from its predecessor.[33] In our case, we see that although v7 is technically more accurate than v6, moving to this version mid-study would mean that operators need to find completely new thresholds to have a similar performance, a process for which there is no straightforward protocol.

Our results, demonstrating the difference in performance by thresholds in versions 6 and 7 of CAD4TB, highlight the importance of optimal threshold selection. The threshold selected influences the sensitivity and specificity of the CAD product, and a product version update without considering the threshold in use could have substantial performance implications.

**Our subgroup analysis demonstrated several findings in relation to both CAD4TB performance and threshold determination.** To our knowledge, we are among few studies to evaluate performance based on hardware,[35] and, to our knowledge, the only study to report threshold differences between the hardware systems included. For CAD4TB v7, different thresholds were needed to achieve the same level of performance across the different X-ray hardware systems. Also, wide AUC confidence intervals were noted across the various hardware due to the limited number of true positives, highlighting the challenge of such data-driven threshold selection. A study exploring the theory and reality of threshold selection highlights that X-ray hardware can be one of the critical factors in threshold selection and should be considered.[9].

Our results showed a statistically significant difference between version 7 and version 6 in the South African group and the group from Lesotho (p = 0.04). It should be noted, however, that the South African cohort all used the Fujifilm hardware, while no group in Lesotho used this machine. Thus, the noted differences and how the populations were selected may also be attributed to the different hardware used.

Both versions of CAD4TB showed lower performance in older age groups (≥60 years) and people with a previous history of TB, observations noted in previous work.[32,36,37] TB causes scarring in the lung tissue of individuals; similarly, older populations are most likely to have lung scarring due to a history of diseases.[10] Thus, lung abnormalities in older people with a TB history are not a definitive indication of active TB; these individuals will, on average, have higher CAD abnormality scores than younger populations. Caution is therefore warranted; implementing a CAD programme in such a setting would flag more of these individuals, thus requiring further confirmatory testing [38].

Our results again emphasise the necessity of understanding the context-specific implications of the threshold selected. A threshold used in one scenario will not be optimal in another.

In our study, both CAD4TB versions showed comparable performance in individuals living with HIV compared to individuals who do not have the virus, in line with one other study. [36] When stratified by sex, there were no noticeable differences in CAD performance between males and females, which aligns with other studies [23,37]. In contrast, another study found lower specificity in males.[36] This study illustrates that CAD performance varies across different populations, versions, and settings, and the optimal triaging threshold varies based on many factors. Using the manufacturer’s standardised threshold abnormality score is questionable, as, similar to other studies, our study showed significant variability in the diagnostic accuracy of the CAD product across the two different settings.,[22,23,32] The recent WHO and the Special Programme for Research and Training in Tropical Disease CAD calibration tool and a recent study by Qin et al. guide how implementers and users can choose the most appropriate threshold specific to their specified population.[11,32] Some studies have identified other approaches to improve CAD performance and accuracy, such as the multivariable model, which predicts the probability of TB [39], and extensive screening methodological approaches to determine optimal threshold selection. [36] A study by Vanobberghen et al. showed that more accurate CAD thresholds can be determined using various approaches that consider even those who have not undergone bacteriological testing. [36] Operational research studies are not always viable in resource-constrained settings like SA and Lesotho; in that case, these various other approaches should be considered.

A strength of our study is that using data from two sources with two different social and demographic characteristics provides more generalisable results. However, the country data is confounded with X-ray hardware and should be interpreted cautiously.

There are limitations to our study. A major limitation is that only participants eligible for testing were included, either because they presented to a health facility (TBT + ) or met further testing criteria (LPS); thus, findings have limited generalisability to use CAD4TB in population screening settings. Our study population was not a typical screening population since in TBT+, only symptomatic individuals at a facility setting were included, and in LPS, only individuals who passed the pre-screening were included. The result is that the dataset consists of a much greater proportion of abnormal CXR images, which results in a higher loss of specificity than a typical screening dataset. Thus, this most likely contributed to the failure to meet these WHO targets. There is a limited set of varying X-ray hardware; therefore, the results may not generalise to all hardware and other settings. Also, some cases and controls in the LPS were bacteriologically confirmed using only Xpert MTB/RIF Ultra, which could lead to false negatives [40]. In conclusion, CAD4TB version 7 showed significant improvement compared to version 6 in populations with a high burden of TB. Additionally, both versions meet the WHO TPP targets in populations below 60 years without a history of TB. Thus, understanding threshold selection and contextually specific settings is paramount to enable wider adoption of this CAD product as an effective triaging and screening tool. The rapid updates of CAD programmes and CAD software versions call for continued investigation and assessment of performance accuracy to ascertain variation and necessary threshold adjustments to ensure the optimal global impact of this AI technology. In addition to performance accuracy investigation, such evaluations enable users to gain an evidence-based understanding of the programmatic implications of these updates, allowing studies to be planned to optimise CAD performance capabilities.

## CRediT authorship contribution statement

**Nonhlanhla Nzimande:** Writing – review & editing, Writing – original draft, Visualization, Validation, Software, Resources, Project administration, Methodology, Investigation, Formal analysis, Data curation, Conceptualization. **Keelin Murphy:** Writing – review & editing, Validation, Supervision, Methodology, Investigation, Formal analysis, Data curation, Conceptualization. **Klaus Reither:** Visualization, Validation, Resources, Project administration, Formal analysis, Data curation, Conceptualization. **Shannon Bosman:** Visualization, Resources, Project administration, Conceptualization. **Irene Ayakaka:** Resources. **Tracy R. Glass:** Writing – review & editing, Formal analysis, Data curation. **Fiona Vanobberghen:** Writing – review & editing, Validation, Formal analysis, Data curation. **Bart K.M. Jacobs:** Writing – review & editing, Validation, Formal analysis, Data curation, Conceptualization. **Aita Signorell:** Writing – review & editing, Visualization. **Jabulani Ncayiyana:** Writing – review & editing, Writing – original draft, Validation, Supervision, Methodology, Formal analysis, Data curation, Conceptualization.

## Declaration of competing interest

The authors declare that they have no known competing financial interests or personal relationships that could have appeared to influence the work reported in this paper.
