# Supplementary material for: Performance of CAD4TB artificial intelligence technology in TB screening programmes among the adult population in South Africa and Lesotho
Source: J Clin Tuberc Other Mycobact Dis. 2025 Jun 4;40:100540. doi: 10.1016/j.jctube.2025.100540 (PMC12179731; doi:10.1016/j.jctube.2025.100540)
Supplement: Supplementary Data 1 [file mmc1.doc]

Supplementary material

**Performance of CAD4TB Artificial Intelligence Technology in TB Screening Programs Among the Adult Population in South Africa and Lesotho**

Table of contents

[**Figure a1** 3](#__RefHeading___Toc192676897)

[**Table a1:** Lesotho Prevalence Survey baseline characteristics 3](#__RefHeading___Toc192676898)

[**Table a2:** TB TRIAGE+ baseline characteristics 4](#__RefHeading___Toc192676899)

[**Figure a2** 5](#__RefHeading___Toc192676900)

[**Figure a3** 5](#__RefHeading___Toc192676901)

[**Figure a4** 5](#__RefHeading___Toc192676902)

[**Table a3:** Detailed subgroup analysis (pairwise comparison) of groups with more than two subgroups 7](#__RefHeading___Toc192676903)

[**Table a4:** Bonferroni multiple testing correction 7](#__RefHeading___Toc192676904)

# **Figure a1**

# **Table a1:** Lesotho Prevalence Survey baseline characteristics

|  | **TB negative**  **5 150, (97.10%)**  **N, (column %)** | **TB positive**  **154, (2.90%)**  **N, (column %)** | | **Total**  **(n 5 304)**  **N, (column %)** | |
| --- | --- | --- | --- | --- | --- |
| **Age (yrs.) Mean, SD** | 50, 19.04 | 55, 18.08 | | 50, 19.03 | |
| **Age group**  15 to <35 years 1 282 (24.89) 24 (15.58) 1 306 (24.62) | | | | |  |
| 35 to <60 years | 2 045 (39.71) | 61 (39.61) | | 2 106 (39.71) | |
| ≥60 years | 1 823 (35.40) | 69 (44.81) | | 1 892 (35.67) | |
| **Sex** |  |  | |  | |
| Male | 2 560 (49.71) | 100 (64.94) | | 2 660 (50.15) | |
| Female  **HIV status**  HIV negative  HIV positive  Unknown  **^a^ Hardware**  Innomed1  Innomed2  Sedecal  **Prev TB**  No  Yes  **CAD4TB**  **^b^** v6 -median, IQR  **^c^** v7- median, IQR | 2 590 (50.29)    3 528 (68.50)  1 152 (22.37)  470 (9.13)  1 842 (35.77)  2 436 (47.30)  872 (16.93*)*    4 323 (83.94)  827 (16.06)    52.31, 17.20  19.60, 21.35 | 54 (35.06)    88 (57.14)  45 (29.22)  21 (13.64)    48 (31.17)  89 (57.79)  17 (11.04)  122 (79.22)  32 (20.78)  74.10, 17.51  55.84, 25.15 | | 2 644 (49.85)    3 616 (68.17)  1 197 (22.57)  491 (9.26)  1 890 (35.63)  2 525 (47.61)  889 (16.76)    4 445 (83.80)  859 (16.20)  52.94, 17.59  20.66, 22.32 | |
|  | | |  | | |

**^a^** Hardware (hardware used in image acquisition) Innomed1=Innomed X-ray machine with Samsung detector, SDR-AGR40CW: SMD4343WS; Innomed2=Innomed X-ray machine with Samsung detector, DGR-RN2N22/WR: SMD4343WS; Sedecal=Sedecal Dragon 5kW Digital X-ray machine with integrated detector

**^b^** CAD4TB v6 (CAD abnormality score)

**^c^** CAD4TB v7 (CAD abnormality score)

# **Table a2:** TB TRIAGE+ baseline characteristics

|  | **TB negative**  **1 086, (89.02%)**  **N, (%)** | **TB positive**  **134, (10.98%)**  **N, (%)** | | **Total**  **(n 1 220)**  **N, (%)** | |
| --- | --- | --- | --- | --- | --- |
| **Age (yrs.) Mean, SD** | 46, 16.11 | 41, 13.98 | | 45, 15.96 | |
| **Age group**  15 to <35 years 310 (28.55) 54 (40.30) 364 (29.84) | | | | |  |
| 35 to <60 years | 526 (48.43) | 61 (45.52) | | 587 (48.11) | |
| ≥60 years | 250 (23.02) | 19 (14.18) | | 269 (22.05) | |
| **Sex** |  |  | |  | |
| Male | 579 (53.31) | 91 (67.91) | | 670 (54.92) | |
| Female  **Country**  South Africa  Lesotho  **HIV status**  HIV negative  HIV positive  Unknown  **^a^ Hardware**  Delft  Fujifilm  **Prev TB**  No  Yes  **CAD4TB**  **^b^** v6 -median, IQR  **^c^** v7- median, IQR | 507 (46.69)    477 (43.92)  609 (56.08)  547 (50.37)  521 (47.97)  18 (1.66)  609 (56.08)  477 (43.92)      823 (75.78)  263 (24.22)    51.65, 20.60  22.08, 25.21 | 43 (32.09)    65 (48.51)  69 (51.49)    55 (41.04)  77 (57.46)  2 (1.49)    69 (51.49)  65 (48.51)  108 (80.60)  26 (19.40)  79.19, 20.87  68.43, 28.34 | | 550 (45.08)    542 (44.43)  678 (55.57)  602(49.34)  598 (49.02)  20 (1.64))  678 (55.57)  542 (44.43)    931 (76.31)  289 (23.69)  54.67, 22.35  27.17, 29.38 | |
|  | | |  | | |

**^a^** Hardware (hardware used in image acquisition) Delft= Delft Light Portable X-ray machine with Canon detector, CXDI Control Software; Fujifilm=FUJIFILM FDR Smart X-ray machine with Vieworks detector, FXRD-1717VA;

**^b^** CAD4TB v6 (CAD abnormality score)

**^c^** CAD4TB v7 (CAD abnormality score)

# **Figure a2**

# **Figure a3**

# **Figure a4**

|  | **CAD4TB v6** |  |  | **CAD4TB v7** |  |
| --- | --- | --- | --- | --- | --- |
| Subgroup | AUC | P-value |  | AUC | P-value |
| **^a^ X-ray Hardware:** |  |  |  |  |  |
| Delft  Fujifilm | 0.81 (95% CI 0.76-0.86)  0.85 (95% CI 0.80-0.91) | 0.26 |  | 0.84 (95% CI 0.79-0.89)  0.91 (95% CI 0.87-0.95) | 0.05 |
| Delft  Innomed1 | 0.81 (95% CI 0.76-0.86)  0.83 (95% CI 0.78-0.87) | 0.68 |  | 0.84 (95% CI 0.79-0.89)  0.83 (95% CI 0.77-0.89) | 0.70 |
| Delft  Innomed2 | 0.81 (95% CI 0.76-0.86)  0.84 (95% CI 0.79-0.89) | 0.41 |  | 0.84 (95% CI 0.79-0.89)  0.87 (95% CI 0.83-0.91) | 0.45 |
| Delft  Sedecal | 0.81 (95% CI 0.76-0.86)  0.77 (95% CI 0.64-0.89) | 0.55 |  | 0.84 (95% CI 0.79-0.89)  0.83 (95% CI 0.71-0.95) | 0.88 |
| Fujifilm  Innomed1 | 0.85 (95% CI 0.80-0.91)  0.83 (95% CI 0.78-0.87) | 0.44 |  | 0.91 (95% CI 0.87-0.95)  0.83 (95% CI 0.77-0.89) | **0.04** |
| Fujifilm  Innomed2 | 0.85 (95% CI 0.80-0.91)  0.84 (95% CI 0.79-0.89) | 0.68 |  | 0.91 (95% CI 0.87-0.95)  0.87 (95% CI 0.83-0.91) | 0.18 |
| Fujifilm  Sedecal | 0.85 (95% CI 0.80-0.91)  0.77 (95% CI 0.64-0.89) | 0.21 |  | 0.91 (95% CI 0.87-0.95)  0.83 (95% CI 0.71-0.95) | 0.25 |
| Innomed1  Innomed2 | 0.83 (95% CI 0.78-0.87)  0.84 (95% CI 0.79-0.89) | 0.68 |  | 0.83 (95% CI 0.77-0.89)  0.87 (95% CI 0.83-0.91) | 0.28 |
| Innomed1  Sedecal | 0.83 (95% CI 0.78-0.87)  0.77 (95% CI 0.64-0.89) | 0.40 |  | 0.83 (95% CI 0.77-0.89)  0.83 (95% CI 0.71-0.95) | 0.94 |
| Innomed2  Sedecal | 0.84 (95% CI 0.79-0.89)  0.77 (95% CI 0.64-0.89) | 0.29 |  | 0.87 (95% CI 0.83-0.91)  0.83 (95% CI 0.71-0.95) | 0.59 |
| **Age groups** |  |  |  |  |  |
| 15 to <35 years  35 to <60 years | 0.90 (95% CI 0.85-0.95)  0.84 (95% CI 0.80-0.88) | **0.04** |  | 0.92 (95% CI 0.88-0.96)  0.88 (95% CI 0.85-0.92) | 0.17 |
| 15 to <35 years  ≥60 years | 0.90 (95% CI 0.85-0.95)  0.77 (95% CI 0.72-0.81) | **P<0.01** |  | 0.92 (95% CI 0.88-0.96)  0.79 (95% CI 0.74-0.84) | **P<0.01** |
| 35 to <60 years  ≥60 years | 0.84 (95% CI 0.80-0.88)  0.77 (95% CI 0.72-0.81) | **0.02** |  | 0.88 (95% CI 0.85-0.92)  0.79 (95% CI 0.74-0.84) | **P<0.01** |

# **Table a3:** Detailed subgroup analysis (pairwise comparison) of groups with more than two subgroups

a Hardware (hardware used in image acquisition) Delft= Delft Light Portable X-ray machine with Canon detector, CXDI Control Software; Fujifilm,=FUJIFILM FDR Smart X-ray machine with Vieworks detector, FXRD-1717VA; Innomed1=Innomed X-ray machine with Samsung detector, SDR-AGR40CW: SMD4343WS; Innomed2=Innomed X-ray machine with Samsung detector, DGR-RN2N22/WR: SMD4343WS; Sedecal=Sedecal Dragon 5kW Digital X-ray machine with integrated detector.

# **Table a4:** Bonferroni multiple testing correction

Bonferroni corrected α for X-ray hardware = α/k (where k is the no. of tests)

= 0.05/ 10

= 0.005

Bonferroni corrected α for age groups = α/k (where k is the no. of tests)

= 0.05/ 3

= 0.017

| **X-ray hardware pairwise comparison** | Bonferroni corrected α (p<0.005)  CAD4TB V6 CAD4TB V7 | |
| --- | --- | --- |
| Delft/Fujifilm | 0.26 | 0.05 |
| Delft/ Innomed1 | 0.68 | 0.70 |
| Delft/ Innomed2 | 0.41 | 0.45 |
| Delft/Sedecal | 0.55 | 0.88 |
| Fujifilm/Innomed1 | 0.44 | 0.04 |
| Fujifilm/Innomed2 | 0.68 | 0.18 |
| Fujifilm/Sedecal | 0.21 | 0.25 |
| Innomed1/Innomed2 | 0.68 | 0.28 |
| Innomed1/Sedecal | 0.40 | 0.94 |
| Innomed2/Sedecal | 0.29 | 0.59 |
| **Age groups pairwise comparison** | Bonferroni corrected α (p<0.017)  CAD4TB V6 CAD4TB V7 | |
| 15 to <35 years/35 to <60 years | 0.04 | 0.17 |
| 15 to <35 years/≥60 years | P<0.01 | P<0.01 |
| 35 to <60 years/≥60 years | 0.02 | P<0.01 |
